# A Rapid Coordinate Transformation Method Applied in Industrial Robot Calibration Based on Characteristic Line Coincidence 

**DOI:** 10.3390/s16020239

**Published:** 2016-02-18

**Authors:** Bailing Liu, Fumin Zhang, Xinghua Qu, Xiaojia Shi

**Affiliations:** State Key Laboratory of Precision Measuring Technology and Instruments, Tianjin University, Tianjin 300072, China; liubailing@tju.edu.cn (B.L.); quxinghua@tju.edu.cn (X.Q.); shixiaojia@tju.edu.cn (X.S.)

**Keywords:** coordinate transformation, robot calibration, photogrammetric system, multi-sensor measurement system

## Abstract

Coordinate transformation plays an indispensable role in industrial measurements, including photogrammetry, geodesy, laser 3-D measurement and robotics. The widely applied methods of coordinate transformation are generally based on solving the equations of point clouds. Despite the high accuracy, this might result in no solution due to the use of ill conditioned matrices. In this paper, a novel coordinate transformation method is proposed, not based on the equation solution but based on the geometric transformation. We construct characteristic lines to represent the coordinate systems. According to the space geometry relation, the characteristic line scan is made to coincide by a series of rotations and translations. The transformation matrix can be obtained using matrix transformation theory. Experiments are designed to compare the proposed method with other methods. The results show that the proposed method has the same high accuracy, but the operation is more convenient and flexible. A multi-sensor combined measurement system is also presented to improve the position accuracy of a robot with the calibration of the robot kinematic parameters. Experimental verification shows that the position accuracy of robot manipulator is improved by 45.8% with the proposed method and robot calibration.

## 1. Introduction

Multi-sensor measurement systems usually have different coordinate systems. The original data must be transformed to a common coordinate system for the convenience of the subsequent data acquisition, comparison and fusion [1,2]. The transformation of coordinate systems is applied in many fields, especially vision measurement and robotics. For example, two images need to have a unified coordinate system for image matching [3]. In camera calibration, the coordinate systems of the image plane and the object plane need to be unified for the inner parameter calculation [4]. In robot systems, the coordinate system of the robot twist needs to be transformed to the tool center position (TCP) to obtain the correct pose of robot manipulators [5,6]. A minor error introduced by an imprecise coordinate transformation could cause problems such as the failure of image matching and track breaking [1]. Especially in an error accumulating system such as series industry robots, the coordinate transformation error would accumulate in each step and thereby decrease the position accuracy of the robot manipulator. Therefore, research on coordinate transformation has been of interest to researchers in recent years.

Industrial robots are well-known to have weak position accuracy compared with their repeatability accuracy. The positioning accuracy degrades with the number of axes of the robotic arm due to error accumulation. Various methods have been presented to improve the position accuracy of robots, such as establishing a kinematic model of the robot and calibrating the kinematic parameters [7]. Denavit and Hartenberg [8] first proposed the D-H model, which was revised to a linear model by Hayati [9]. It provides the basis for the kinematic calibration. Due to the geometry and non-geometry errors of the robot, the traditional robot self-calibration method based on the D-H model cannot accurately describe the robot pose. To avoid the influence of the robot body, many researchers have utilized external measuring instruments to calibrate the robot online [10,11]. To achieve the aim of calibration, the primary process is to unify the coordinate systems of a calibrated instruments and the robot. Only in this way is it possible to use the measurement results to correct the kinematic parameters of the robot. With an inaccurate coordinate transformation method, the transformation error might merge into the revised kinematics parameters, thereby failing to improve the positioning accuracy of the robot through the calibration of kinematic parameters. Therefore, an accurate method of coordinate transformation is indispensable in the field of robot calibration. The well-developed and widely-used methods of coordinate transformation at present might be classified into several categories: the Three-Point method, Small-Angle Approximation method, Rodrigo Matrix method, Singular Value Decomposition (SVD) method, Quaternion method and Least Squares method [2]. The Three-Point method uses three non-collinear points in space to construct an intermediate reference coordinate system [12,13]. The transformation relationship between the initial coordinate system and the target coordinate system is obtained by their relationship relative to the intermediate reference coordinate system. Depending on the choice of the public points, the accuracy of the Three-Point method might be unstable. The Small-Angle Approximation method means that the rotation matrix can be simplified by using the approximate relationship of a trigonometric function (sinθ=θ, cosθ=1) when the angle between the two coordinate systems is small (less than 5°). It is more suitable for the coordinate transformation of small angles. The Rodrigo matrix is a method of constructing a rotation matrix by using the anti-symmetric matrix [14,15]. Despite its high accuracy and good stability, the algorithm might be complex and difficult. The Singular Value Decomposition method (SVD) is a matrix decomposition method that can solve the minimization of the objective function based on the minimum square error sum [16]. The method is accurate and easy to implement, but it might be difficult to work out the rotation matrix under a dense public point cloud. The Quaternion method uses four element vectors ($q_{0},q_{1},q_{2},q_{3}$) to describe the coordinate rotation matrix [17,18]. The aim of the algorithm is to solve for the maximum eigenvalue and the corresponding feature vector when the quadratic is minimized. It is a simple and precise method, but there might be no solution due to the use of ill conditioned matrices. In practice, complex calculations and unstable results would make the application more difficult and complicated. Therefore, researchers are searching for a simpler and more stable method of coordinate transformation. For example, Zhang *et al.* proposed a practical method of coordinate transformation in robot calibration [19]. This method rotates three single axes of the robot to calculate the normal vectors in three directions, combined with the data of the calibration sensor. Then, combined with the own readings of the robot, the rotation matrix and translation matrix are obtained. The method avoids the need to solve an equation and complex calculations, but it might be affected by any manufacturing errors of the robot and requires a calibration sensor with a large measuring range that can cover the full working range of the robot.

## 2. Online Calibration System of Robot

**Figure 1:**
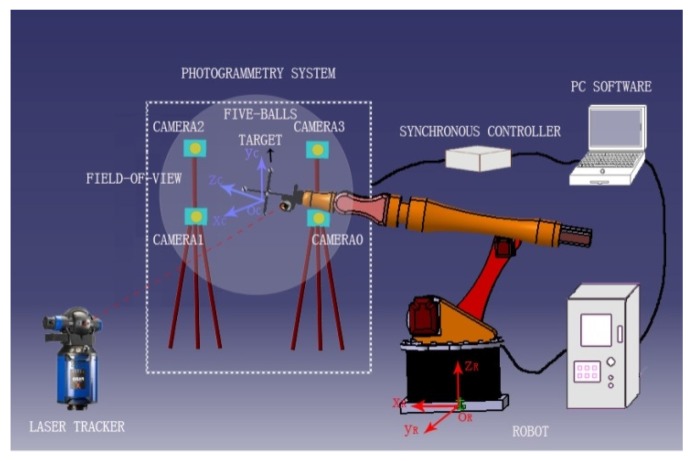
Online calibration system of robot kinematic parameters.

Industrial robots have the characteristics of high repeatability positioning accuracy and low absolute positioning accuracy. This is due to the structure of the robot, manufacturing errors, kinematic parameter error and environmental influence [10]. To improve the absolute positioning accuracy of the robot, the use of an external sensor to measure the position of the robot manipulator it any effective approach. This paper proposes an on-line calibration system for the kinematic parameters of the robot using a laser tracker and a close-range photogrammetric system, as Figure 1 shows. According to the differential equations constructed by the kinematic parameters of each robot axis, the final mathematic model of kinematic parameters of the robot is established. The position errors of the robot manipulator are obtained by comparing the coordinates in the robot base coordinate system and the measurement sensor system. Then, the errors, including the coordinate transformation error, target installation error and position and angle errors of the robot kinematic parameters, are separately corrected. In the robot calibration, on the one hand, the coordinate transformation error directly affects the final error correction of the kinematic parameters. On the other hand, the coordinate systems of sensors are often required to transform in the on-line combined measurement system. Therefore, the premise of obtaining the position errors of a robot manipulator is to unify the coordinate systems of the various measurement sensors by an accurate, fast and stable coordinate transformation algorithm.

In combination with the characteristics of the robot, we propose a practical coordinate transformation method. It extracts the characteristic lines from the point clouds in different coordinate systems. According to the theory of space analytic geometry, the rotation and translation parameters needed for the coincidence of the characteristic line scan be calculated. Then, the coordinate transformation matrix is calculated. The coincidence of the characteristic lines represents the coincidence of the point clouds as well as the coincidence of the two coordinate systems. 

This method has some advantages. First, it does not require the solution of equations and complex calculations. Second, because the transformation matrix is obtained from the space geometry relationships, it would not be affected by robot errors or other environmental factors. The result is accurate and stable. Third, it does not require a sensor with a large field of view. Fourth, the algorithm is small and fast without occupying processor time and resources, and can be integrated into the host computer program. It could be applied easily in measurement coordinate systems that often need to change. 

## 3. Methods of Online Calibration System

### 3.1. Method of Coordinate Transformation

Suppose that S is a cubic point cloud in space. Point cloud M is the form of S located in the coordinate system of the sensor *O_S_X_S_Y_S_Z_S_*. N is the form of S located in the robot base coordinate system *O_r_X_r_Y_r_Z_r_*. M' represents the point cloud M transformed from the coordinate system of the sensor *O_S_X_S_Y_S_Z_S_* to the robot base coordinate system *O_r_X_r_Y_r_Z_r_* with the transformation matrix *T_S_^r^*. The difference between N and M' is the transformation error caused by the transfer matrix *T_S_^r^*. Then, the coincidence of the coordinate systems *O_S_X_S_Y_S_Z_S_* and *O_r_X_r_Y_r_Z_r_* can be expressed as the coincidence of the two point clouds N and M'. For simplifying this mathematical model of the transformation process, we establish several characteristic lines instead of each point cloud. As verified by experiment, at least two characteristic lines are required to ensure the transformation accuracy. 

**Figure 2:**
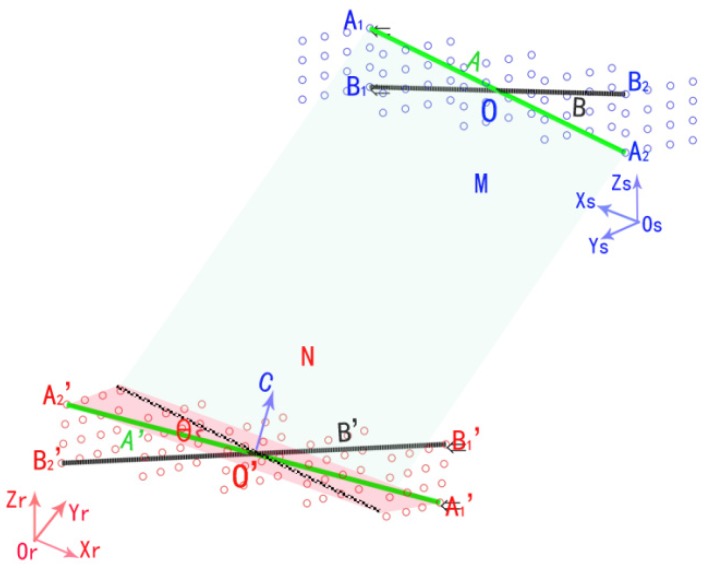
The schematic diagram of coordinate transformation method.

**Figure 3:**
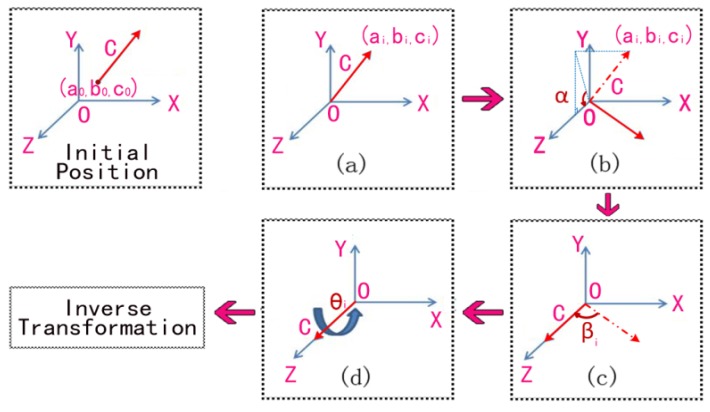
Schematic diagram of a vector rotated around an arbitrary axis.

In Figure 2, two points A_1_ and A_2_ are chosen to be linked to the characteristic line A. Points B_1_ and B_2_ form characteristic line B. Similarly, in point cloud N, the corresponding points A_1_'and A_2_' form line A', and points B_1_' and B_2_' form line B'. To achieve the coincidence of lines A and A', line A must be rotated around an axis in space. The rotated axis is the vector C which is perpendicular to the plane constructed by lines A and A'. As Figure 3 shows, the process of a vector rotating around an arbitrary axis can be divided into a series of rotations around the axis X, Y, Z. The following are the decomposition steps.

Take the first coincidence of Lines A and A' as an example:

(a) Translate the rotation axis to the coordinate origin. The corresponding transformation matrix can be calculated as:
  (1) $$T(x_{1},y_{1},z_{1})=\left[\begin{array}{cccc} 1 & 0 & 0 & -a_{0}\\ 0 & 1 & 0 & -b_{0}\\ 0 & 0 & 1 & -c_{0}\\ 0 & 0 & 0 & 1 \end{array}\right]$$
  where, (*a_0_*, *b_0_*, *c_0_*) is the coordinates of the center point of line A.
(b) Rotate the axis α_1_ degrees to Plane XOZ.
  (2) $$Rx(\alpha_{1})=\left[\begin{array}{cccc} 1 & 0 & 0 & 0\\ 0 & \cos\alpha_{1} & -\sin\alpha_{1} & 0\\ 0 & \sin\alpha_{1} & \cos\alpha_{1} & 0\\ 0 & 0 & 0 & 1 \end{array}\right]$$
  $\alpha_{1}$ is the angle between the axis and plane XOZ. It can be obtained by $\cos\alpha_{1}=\frac{c_{1}}{\sqrt{{b_{1}}^{2}+{c_{1}}^{2}}}$, $\sin\alpha_{1}=\frac{b_{1}}{\sqrt{{b_{1}}^{2}+{c_{1}}^{2}}}$, where, (*a_1_*, *b_1_*, *c_1_*) are the coordinates of vector *C*, as Figure 3b shows.
(c) Rotate the axis β_1_ degrees to coincide with Axis Z.
  (3) $$Ry(-\beta_{1})=\left[\begin{array}{cccc} \cos\beta_{1} & 0 & \sin(-\beta_{1}) & 0\\ 0 & 1 & 0 & 0\\ -\sin(-\beta_{1}) & 0 & \cos\beta_{1} & 0\\ 0 & 0 & 0 & 1 \end{array}\right]$$
  where, $\beta_{1}$ is the angle between the rotation axis and axis Z. It can be obtained by $\left\{ \begin{array}{l} \cos(-\beta_{1})=\cos\beta_{1}=\frac{\sqrt{{b_{1}}^{2}+{c_{1}}^{2}}}{\sqrt{{a_{1}}^{2}+{b_{1}}^{2}+{c_{1}}^{2}}}\\ \sin(-\beta_{1})=-\sin\beta_{1}=-\frac{a_{1}}{\sqrt{{a_{1}}^{2}+{b_{1}}^{2}+{c_{1}}^{2}}} \end{array}\right.$.
(d) Rotate the axis $\theta_{1}$ degrees around Axis Z, as shown in Figure 3d.
  (4) $$Rz(\theta_{1})=\left[\begin{array}{cccc} \cos\theta_{1} & \sin\theta_{1} & 0 & 0\\ -\sin\theta_{1} & \cos\theta_{1} & 0 & 0\\ 0 & 0 & 1 & 0\\ 0 & 0 & 0 & 1 \end{array}\right]$$
  where $\theta_{1}$ is the angle between lines A and A', which can be obtained by $\theta_{1}=<\vec{A},\vec{A'}>=arccos(\frac{\vec{A}\cdot \vec{A'}}{\left|\vec{A}\right|\left|\vec{A'}\right|})$.
(e) Rotate the axis by reversing the process of Step (c)
  (5) $$Ry(\beta_{1})=\left[\begin{array}{cccc} \cos\beta_{1} & 0 & -\sin\beta_{1} & 0\\ 0 & 1 & 0 & 0\\ \sin\beta_{1} & 0 & \cos\beta_{1} & 0\\ 0 & 0 & 0 & 1 \end{array}\right]$$
  where, $\beta_{1}$ is as the same as in step (c).
(f) Rotate the axis by reversing the process of Step (b).
  (6) $$Rx(-\alpha_{1})=\left[\begin{array}{cccc} 1 & 0 & 0 & 0\\ 0 & \cos\alpha_{1} & \sin\alpha_{1} & 0\\ 0 & -\sin\alpha_{1} & \cos\alpha_{1} & 0\\ 0 & 0 & 0 & 1 \end{array}\right]$$
  where, $\alpha_{1}$ is as the same as in step (b).
(g) Rotate the axis by reversing the process of Step (a)
  (7) $$T(-x_{1},-y_{1},-z_{1})=\left[\begin{array}{cccc} 1 & 0 & 0 & a_{0}\\ 0 & 1 & 0 & b_{0}\\ 0 & 0 & 1 & c_{0}\\ 0 & 0 & 0 & 1 \end{array}\right]$$
  where, (*a_0_*, *b_0_*, *c_0_*) is as the same as in step (a).

Combining all of the previous steps, the final transformation matrix $Trt_{1}$ of the first parallel (lines A and A') is expressed as:

(8) $$Trt_{1}=T(-x_{1},-y_{1},-z_{1})\cdot Rx(-\alpha_{1})\cdot Ry(\beta_{1})\cdot Rz(\theta_{1})\cdot Ry(-\beta_{1})\cdot Rx(\alpha_{1})\cdot T(x_{1},y_{1},z_{1})$$

Through the rotation matrix Trt_1_ calculated by Equation (8), the points P_i_(x, y, z) in point cloud M can generate a new point cloud M_1_ by Equation (9).

(9) Pi'(x,y,z)=Trt⋅Pi(x,y,z)

**Figure 4:**
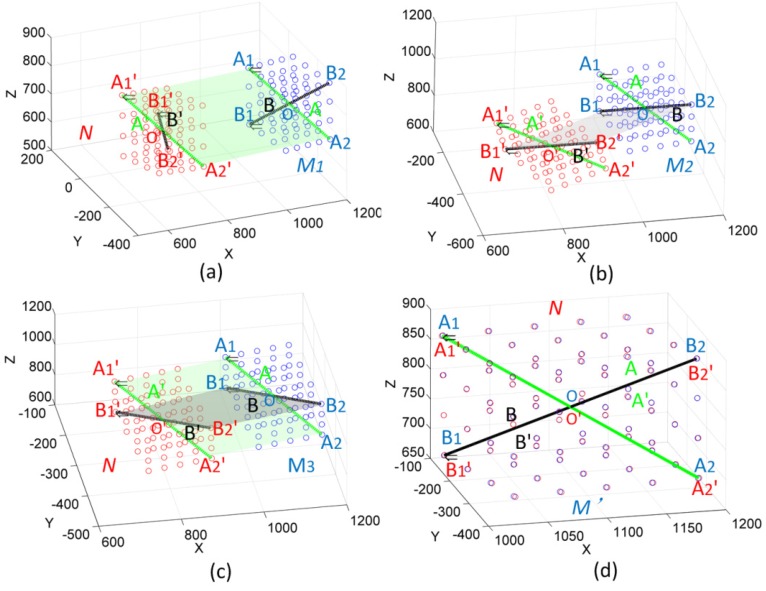
The processes of the coordinate transformation method.

Then, the characteristic line A of the new point cloud M_1_ is parallel with the characteristic line A' of point cloud N, as Figure 4a shows.

Based on the new point cloud M_1_ and point cloud N, the rotation matrix Trt_2_, which make the Line B of Point cloud M_1_ parallel with Line B' of Point cloud N, can be calculated through Equations (1)–(8):

(10) $$Trt_{2}=T(-x_{2},-y_{2},-z_{2})\cdot Rx(-\alpha_{2})\cdot Ry(\beta_{2})\cdot Rz(\theta_{2})\cdot Ry(-\beta_{2})\cdot Rx(\alpha_{2})\cdot T(x_{2},y_{2},z_{2})$$

Through the rotation matrix Trt_2_, the points P_i_(x, y, z) in point cloud M_1_ can generate a new point cloud M_2_ again by Equation (9). Then, the characteristic line B of the new point cloud M_2_ is parallel with the characteristic line B' of point cloud N, as Figure 4b shows. 

Since the point clouds are cubic, the characteristic lines are the diagonal lines. So, B⊥A, B'⊥A'. Since, B//B'. Then, B⊥A', B'⊥A. Therefore, the parallel Line B and B' are perpendicular to Line A and A'. There is an angle *θ* between Line A of point cloud M_2_ and Line A' of point cloud N, so the Line B of point cloud M_2_ is chosen as the rotation axis. The angle between Line A of point cloud M_2_ and Line A' of point cloud N is chosen as the rotation angle. The point cloud M_2_ is rotated by the above parameters. Then, the Line A of point cloud M_2_ and Line A' of point cloud N are parallel, like Line B of point cloud M_2_ and Line B' of point cloud N. Similarly, the rotation matrix Trt_3_ can be calculated by Equations (1)–(8):

(11) $$Trt_{3}=T(-x_{3},-y_{3},-z_{3})\cdot Rx(-\alpha_{3})\cdot Ry(\beta_{3})\cdot Rz(\theta_{3})\cdot Ry(-\beta_{3})\cdot Rx(\alpha_{3})\cdot T(x_{3},y_{3},z_{3})$$

The points P_i_(x,y,z) in point cloud M_2_ can generate a new point cloud M_3_ by Equation (9), which are parallel with the point cloud N, as Figure 4c shows. In order to make coincident the point cloud M_3_ and point cloud N, the translation matrix *Tr* needs to be calculated by the two center points of Line A and A'. The new point cloud M' can be generated after translated by *Tr*. Therefore, through a series of simple rotations and translation, the two point clouds N and M' are coincident, as Figure 4d shows. The final transformation matrix is shown as Equation (12). The result, as a necessary preparation step, can then be used in robot calibration:

(12) $$Trt=Trt_{3}\cdot Trt_{2}\cdot Trt_{1}+Tr$$

### 3.2. Method of Robot Calibration

The actual kinematic parameters of the robot deviate from their nominal values, which is referred to as kinematic errors [10]. The kinematic parameter calibration of a robot is an effective way to improve the absolute position accuracy of the robot manipulator. A simple robot self-calibration method based on the D-H model is described as follows. Reference [20] gives a more detailed description.

Assume that $B_{p}=\left[\begin{array}{cccc} r_{1p} & r_{2p} & r_{3p} & p_{xp}\\ r_{4p} & r_{5p} & r_{6p} & p_{yp}\\ r_{7p} & r_{8p} & r_{9p} & p_{zp}\\ 0 & 0 & 0 & 1 \end{array}\right]$ is the pose of a certain point in the coordinate system of the photogrammetric system, where $r_{1p}\sim {r_{9}}_{p}$ are the attitude parameters and $p_{xp}\sim p_{zp}$ are the position parameters. Through transformation from the coordinate system of the measurement sensor $O_{p}X_{p}Y_{p}Z_{p}$ to the robot base coordinate system $O_{o}X_{o}Y_{o}Z_{o}$, the point pose $B_{o}=\left[\begin{array}{cccc} r_{1o} & r_{2o} & r_{3o} & p_{xo}\\ r_{4o} & r_{5o} & r_{6o} & p_{yo}\\ r_{7o} & r_{8o} & r_{9o} & p_{zo}\\ 0 & 0 & 0 & 1 \end{array}\right]$ can be obtained by Equation (12):

(13) $$B_{o}=Trt\times B_{p}$$

where, *Trt* is the transformation matrix, which can be obtained by the method described in Section 3.1.

Given the six DOF robot in the lab, the transformation matrix from the robot tool coordinate system to the robot base coordinate system is expressed as:

(14) $$T_{0}^{N}=T_{0}^{1}T_{1}^{2}LT_{n-1}^{n}LT_{N-1}^{N}\;\left(N=6\right)$$

In this system, the cooperation target of the measurement sensor, which is set up at the end axis of the robot, should be considered as an additional axis, Axis 7. Then, the transformation matrix from Axis 6 to Axis 7 is:

(15) $$T_{6}^{7}=\left[\begin{array}{cccc} 1 & 0 & 0 & t_{x}\\ 0 & 1 & 0 & t_{y}\\ 0 & 0 & 1 & t_{z}\\ 0 & 0 & 0 & 1 \end{array}\right]$$

where $t_{x},t_{y},t_{z}$ are the translation vectors, which can be measured previously. Therefore, according to the kinematic model of the robot, the typical coordinates of the robot manipulator in the robot base coordinate system $O_{O}X_{O}Y_{O}Z_{O}$ is expressed as:

(16) $$B_{o}=\left(\sum_{i=1}^{7}T_{i-1}^{i}\right)\cdot B_{t}$$

where $B_{t}$ is the point pose in the robot tool coordinate system, and $B_{o}$ is the point pose from the robot tool coordinate system to the robot base coordinate system. 

In the robot calibration, the kinematic parameters are the most significant impact factors, which usually means the link parameters of the robot. In the D-H model, the link parameters include the length of the link a, the link angle α, the joint displacement d and the rotation angle of the joint θ. With the disturbances of the four link parameters, the position error matrix for adjacent robot axes $dT_{i-1}^{i}$ can be expressed as:

(17) $$dT_{i-1}^{i}=\frac{\partial T_{i-1}^{i}}{\partial \theta_{i}}\Delta \theta_{i}+\frac{\partial T_{i-1}^{i}}{\partial \alpha_{i}}\Delta \alpha_{i}+\frac{\partial T_{i-1}^{i}}{\partial a_{i}}\Delta a_{i}+\frac{\partial T_{i-1}^{i}}{\partial d_{i}}\Delta d_{i}$$

where $\Delta \theta_{i}$,$\Delta \alpha_{i}$,$\Delta a_{i}$ and $\Delta d_{i}$ are the small errors of link parameters. Suppose that $A_{qi}={\left(T_{i-1}^{i}\right)}^{-1}\cdot \frac{\partial T_{i-1}^{i}}{\partial q_{i}}$, where, q represents the link parameters (a, d, α, θ). 

If every two adjacent axes are influenced by the link parameters, the transformation matrix from the robot base coordinate system to the coordinate system of the robot manipulator can be expressed as:

(18) $$T_{0}^{N}+dT_{0}^{N}=\prod_{i=1}^{N}\left(T_{i-1}^{i}+dT_{i-1}^{i}\right)=\prod_{i=1}^{N}\left(T_{i-1}^{i}+T_{i-1}^{i}\Delta_{i}\right)\;\left(N=6\right)$$

where, $T_{0}^{N}$ is the typical transformation matrix from the robot base coordinate system to the coordinate system of the robot manipulator and $dT_{0}^{N}$ is the error matrix caused by the link parameters. Through expanding $dT_{0}^{N}$ and performing a large number of simplifications and combinations, Equation (18) can be simplified as:

(19) $$\begin{array}{l} dT_{0}^{N}=T_{0}^{1}A_{\theta 1}T_{1}^{N}\Delta \theta_{1}+T_{0}^{1}A_{\alpha 1}T_{1}^{N}\Delta \alpha_{1}+T_{0}^{1}A_{a1}T_{1}^{N}\Delta a_{1}+T_{0}^{1}A_{d1}T_{1}^{N}\Delta d_{1}\\ +T_{0}^{2}A_{\theta 2}T_{2}^{N}\Delta \theta_{2}+T_{0}^{2}A_{\alpha 2}T_{2}^{N}\Delta \alpha_{2}+T_{0}^{2}A_{a2}T_{2}^{N}\Delta a_{2}+T_{0}^{2}A_{d2}T_{2}^{N}\Delta d_{2}\\ +L+T_{0}^{N}A_{\theta N}\Delta \theta_{N}+T_{0}^{N}A_{\alpha N}\Delta \alpha_{N}+T_{0}^{N}A_{aN}\Delta a_{N}+T_{0}^{N}A_{dN}\Delta d_{N} \end{array}$$

Suppose that $k_{iq}=T_{0}^{i}A_{qi}T_{1}^{N}$, where, q represents the four link parameters. The position error of the robot manipulator can be simplified as given in Equation (20):

(20) $$\begin{array}{l} \Delta p={\left[d_{tx}d_{ty}d_{tz}\right]}^{T}\\ =\left[\begin{array}{cccccccccc} k_{1\theta }^{x} & k_{1\alpha }^{x} & k_{1a}^{x} & k_{1d}^{x} & k_{2\theta }^{x} & L & k_{6\theta }^{x} & k_{tx}^{x} & k_{ty}^{x} & k_{tz}^{x}\\ k_{1\theta }^{y} & k_{1\alpha }^{y} & k_{1a}^{y} & k_{1d}^{y} & k_{2\theta }^{y} & L & k_{6\theta }^{y} & k_{tx}^{y} & k_{ty}^{y} & k_{tz}^{y}\\ k_{1\theta }^{z} & k_{1\alpha }^{z} & k_{1a}^{z} & k_{1d}^{z} & k_{2\theta }^{z} & L & k_{6\theta }^{z} & k_{tx}^{z} & k_{ty}^{z} & k_{tz}^{z} \end{array}\right]\cdot \\ {\left[\Delta \theta_{1}\Delta \alpha_{1}\Delta a_{1}\Delta d_{1}\Delta \theta_{2}\cdots \Delta d_{6}\Delta t_{x}\Delta t_{y}\Delta t_{z}\right]}^{T}\\ =B_{i}\Delta q_{i} \end{array}$$

where, Δp is the position error of the robot manipulator. $d_{tx},d_{ty},d_{tz}$ are the Cartesian coordinate components of the position error and $B_{i}=\left[\begin{array}{ccc} k_{1\theta }^{x} & L & k_{tz}^{x}\\ M & O & M\\ k_{1\theta }^{z} & L & k_{tz}^{z} \end{array}\right]$ is the parameter matrix related to the typical position value of the robot manipulator. In this paper, because the DOF of the series robot is 6, $\Delta q_{i}=[\Delta \theta_{1}\sim \Delta t_{z}]$ includes 24 kinematics parameters of the robot $a_{1}\sim a_{6}$, $d_{1}\sim d_{6}$, $\alpha_{1}\sim \alpha_{6}$, $\theta_{1}\sim \theta_{6}$ and three translation error variables of *T*_6_^7^. Therefore, there are 27 parameters of the robot that need to be calibrated. In Equation (20), the left side of equation is the position error at each point, as measured by the measurement sensor, and the right side is the kinematics errors that need to be corrected. These errors can be revised by the least squares method in the generalized inverse matrix sense. 

## 4. Experiments and Analysis

Through the designed experiments, we show how to use the proposed coordinate transformation method to achieve the coordinate transformation of the on-line robot calibration system. Using verification experiments, we determine the result of the robot calibration using the proposed method. For evaluating the performance of the proposed method, it is compared with four other common methods of coordinate transformation under the same experimental conditions. 

### 4.1. Coordinate Transformationin an On-line Robot Calibration System

The on-line robot calibration system we constructed includes an industrial robot, a photographic system and a laser tracker as shown in Figure 4. The model of the robot in lab is the KR 5 arc from KUKA Co. Ltd. (Augsburg, Germany), one of the world's top robotic companies. Its arm length is 1.4 m and the working envelope is 8.4 m^3^. For covering most of the robot working range, the close range photogrammetric system in the lab, TENYOUN 3DMoCap-GC130 (Beijing, China), requires a field of view of more than 1 m× 1 m× 1 m without any dead angle. To achieve the goal of on-line measurement, a multi-camera system is needed. We used a multi-camera system symmetrically formed by four CMOS cameras with fixed focal lengths of 6 mm. The laser tracker in the lab, FARO Xi from FARO Co, Ltd. (Lake Mary, FL, USA) is a well-known high accuracy instrument whose absolute distance measurement (ADM) is 10 μm ± 1.1 μm/mL. The laser beam can easily be lost in tracking because of barriers or the acceleration of the target, which would cause minor errors. Therefore, we combine the laser tracker with the photographic system to improve the measurement accuracy and stability and thereby make full use of the advantages of the high accuracy of the laser tracker and the free light-of-sight of the photographic system. After proper data fusion, the two types of data from the photographic system and the laser tracker can be gathered together. The method of data fusion and the verified experimental result are detailed in reference [21]. In the experiment, 80 points in the public field of the robot and the photogrammetric system are picked to build a cube of 200 mm × 200 mm × 200 mm. The reason for building a cube is to facilitate the selection of characteristic lines and the calculation of coincidence parameters. The two targets of the photogrammetric system and laser tracker are installed together with the end axis of the robot by a multi-faced fixture. To obtain accurate and stable data, the robot stops for 7 s at each location, and the sensors measure each point 20 times, providing an adequate measurement time for the photographic system and laser tracker. The experimental parameters of the photogrammetric system are an exposure time of 15 us, a frequency of 10 fps and a gain of 40, based on experience.

According to Equations (1)–(7) and the experimental data, we can obtain the parameters of the transformation matrix shown in Table 1, where *a_i_*–*θ_i_*. are the parameters for the coincidence of characteristic lines in Equations (1)–(7).

According to Equations (8)–(10), the transformation matrices from the robot base coordinate system to the coordinate system of the sensors are calculated as:

$$Trt_{rp}=\left[\begin{array}{cccc} 0.99917951 & 0.03370790 & -0.02245170 & 1003.54380\\ 0.03352107 & -0.99940061 & -0.00864662 & 167.88234\\ -0.02272970 & 0.00788692 & -0.99971054 & 984.54935\\ 0 & 0 & 0 & 1 \end{array}\right]\;Trt_{rl}=\left[\begin{array}{rrrr} 0.999178 & 0.033635 & -0.02262 & -832.501\\ 0.033819 & -0.9994 & 0.007803 & 131.4773\\ -0.02235 & -0.00856 & -0.99971 & 1004.76\\ 0 & 0 & 0 & 1 \end{array}\right]$$

where, $Trt_{rp}$ is the transformation matrix from the robot base coordinate system to the coordinate system of the photogrammetric system. $Trt_{rl}$ is the transformation matrix from the robot base coordinate system tothe coordinate system of the laser tracker.

By means of the above transformation matrix, we can obtain the point cloud coordinates transformed from the coordinate system of the robot to that of the sensors by Equation (12). Both the origin coordinates before and after transformation as well as the transformation error are shown in Table 2, where, *P_x_*,*P_y_*,*P_z_* and *R_x_*,*R_y_*,*R_z_* are three components of the original coordinates in two different coordinate systems. *T_x_*,*T_y_*,*T_z_* are the coordinates of points transformed from the robot base coordinate system to the sensor coordinate system, and Δx,Δy,Δz are the three components of the transformation error. 

**Figure 5:**
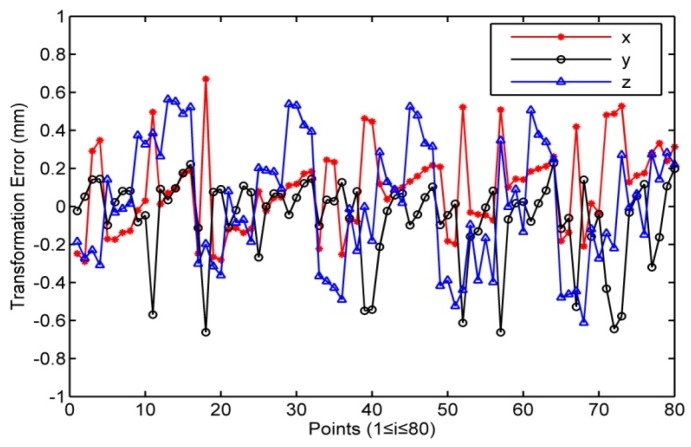
Transformation error from robot to photogrammetric system.

**Figure 6:**
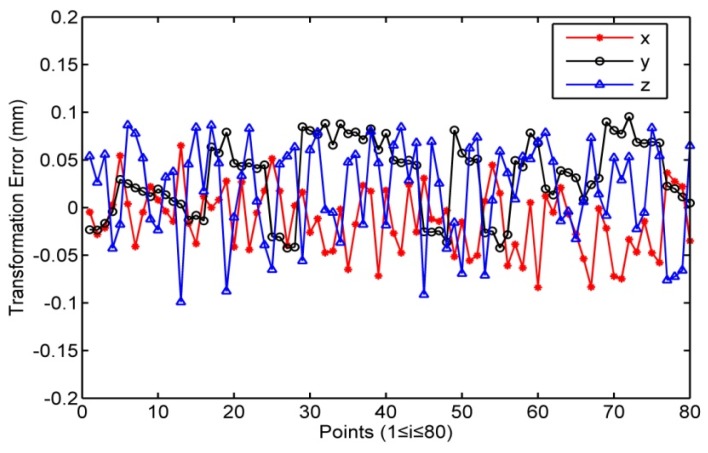
Transformation error from robot to laser tracker.

It is can be calculated from Table 2 that the average values of the transformation error between the coordinate systems of the robot and photogrammetric system are $\bar{\Delta x}$ = 0.106 mm, $\bar{\Delta y}$ = −0.062 mm and $\bar{\Delta z}$ = 0.013 mm. The average values of the transformation error between the coordinate systems of the robot and laser tracker are $\bar{\Delta x}$ = −0.015 mm, $\bar{\Delta y}$ = 0.041 mm and $\bar{\Delta z}$ =0.023 mm. Figure 5 and Figure 6 show that the transformation error of the photogrammetric system is approximately 10 times greater than that of the laser tracker. As in the earlier presentation, the nominal measurement accuracy of the photogrammetric system is 10^−2^ mm and that of the laser tracker is 10^−3^ mm. The results illustrate that the transformation accuracy has the same order of magnitude as that of the measurement sensor. This indicates that the transformation error is so small that it would not influence the accuracy of the sensors. The transformation method can also make the error distribution of the low precision sensor more uniform to improve the transformation accuracy and the accuracy of the robot calibration.

### 4.2. Position Error of Robot after Coordinate Transformation and Calibration

**Figure 7:**
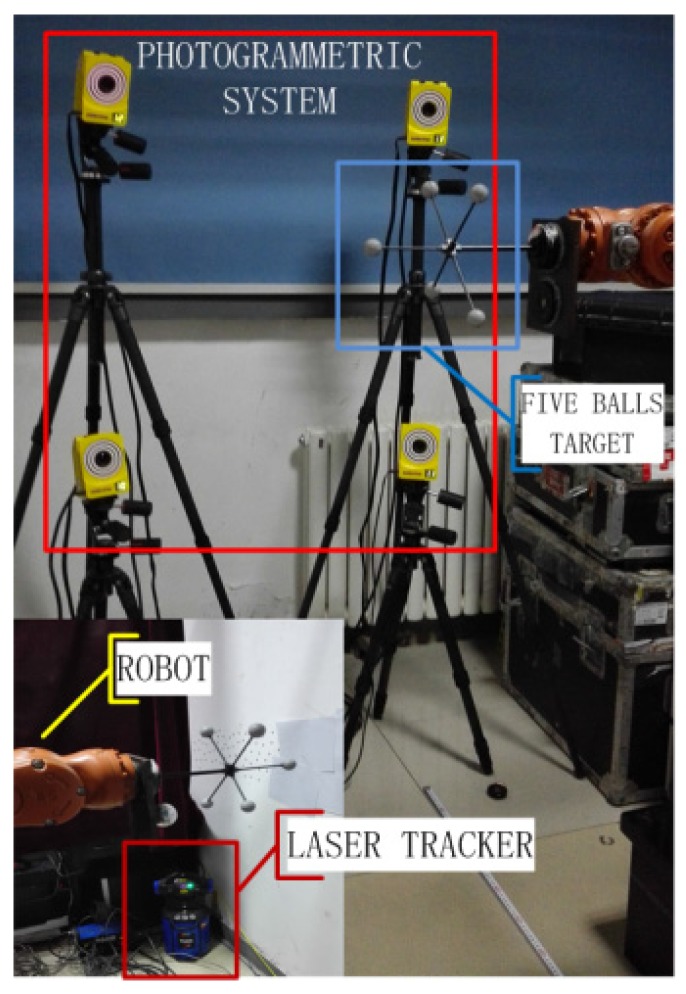
Measurement system.

**Figure 8:**
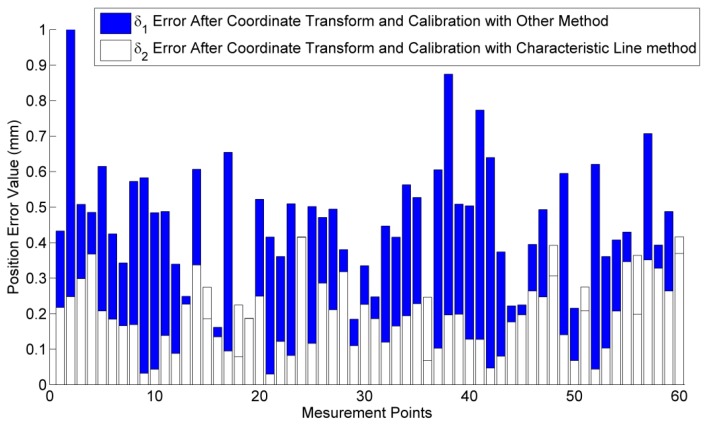
Position errors after robot calibration.

Experiments are designed to calibrate the kinematic parameters of the robot. The measurement system is shown in Figure 7. Sixty points in space are used for the calibration. After the coordinate transformation, the position errors between the sensor and the robot manipulator are obtained. A constraint method based on the minimum distance error is adopted to calibrate the robot kinematic parameters [20,21]. Twenty seven kinematic parameters, including 24 link parameters and three parameters of the fixture, are corrected. Then, 60 correct positions are calculated using the calibrated robot kinematic parameters. To evaluate the performance of the proposed method, we use a group of calibration results, which adopts a different coordinate transformation method and the same robot calibration algorithm, as a comparison. The position errors of the robot after the coordinate transformation and calibration are shown in Figure 8. *δ_1_* is the position error after the coordinate transformation with the other method [19], and *δ_2_* is the position error after the coordinate transformation with the proposed method (Characteristic Line method).

In position measurement, the distance between two points is often used to evaluate the position accuracy, which is called the root mean square (RMS) error expressed by:

(21) $$RMS_{d_{i}}=\sqrt{{\left({x_{i}}^{'}-x_{i}\right)}^{2}+{\left({y_{i}}^{'}-y_{i}\right)}^{2}+{\left({z_{i}}^{'}-z_{i}\right)}^{2}}$$

It can be indicated from Figure 5 that the average RMS using the other coordinate transformation method is $\bar{\delta_{1}}=0.436$ mm. while the average RMS using the proposed method is $\bar{\delta_{2}}=0.200$ mm. The position accuracy is improved by 45.8% using the Characteristic Line method.

To evaluate the accuracy distribution of the robot for different areas of the working range, a new set of testing data are utilized in a demonstration experiment. The coordinates of the center of robot calibration region O is (750, 0, 1000) in the robot base coordinate system. Taking O as the center of the circle, 200 mm as the radius, in this region the positioning accuracy of the robot would be the highest. To verify the distribution of the robot accuracy in the non-calibration regions, five positions O_1_ (1000, 150, 550), O_2_ (780, 710, 900), O_3_ (780, 870, 410), O_4_ (600, −800, 860), O_5_ (940, −960, 450) are chosen. Taking the five positions as the centers of the circle, 200 mm as the radius, in each region 60 points are chosen to calibrate robot as same as the previous calibration experiment. The position errors in different regions are shown in Table 3.

It is indicated from Table 3 that the average RMS of robot position error within the calibration region is 0.200 mm. The position error outside the calibration region is about 0.323 mm. It is proved that the calibration accuracy isn't consistent in the whole working range of robot. Therefore, this calibration method is more applicable to a smaller working range of the robot.

### 4.3. Accuracy of Coordinate Transformation Method

To obtain the accuracy of the proposed coordinate transformation method, an experiment is designed using the laser tracker. The laser tracker is placed at two different stations to measure five common points. These common points should be collinear; otherwise, the Jacobi matrix will have a rank defect of one and be singular. The choice of five points is for improving the transformation accuracy. After the unification of the coordinate systems by the proposed method, the measurement results of the laser tracker are compared. Then, the accuracy of the proposed transformation method (Characteristic Line method) can be calculated, as Table 3 shows.

In addition, for evaluating the performance of the proposed method, we calculate the transformation accuracy of four methods on the same points: the Three Point method, Rodrigo Matrix method, Singular Value Decomposition method and Quaternion method. The measurement data of five public points measured by the laser tracker from two different stations are substituted into the four algorithms. Then, the rotation matrix R and translation matrix *T* can be calculated, and the coordinate transformation matrix *Trt* are also obtained. According to Equation (22), the new coordinates after transformation by the *Trt* can be generated as below:

(22) (xi'yi'zi')s2=R(xiyizi)s1+T=(r11r12r13r21r22r23r31r32r33)(xiyizi)s1+(x0y0z0)

where (*x_i_*, *y_i_*, *z_i_*)*_s1_* is the coordinate of the ith point measured by the laser tracker at Station 1. (*x_i_'*, *y_i_'*, *z_i_'*)*_s2_* are the new coordinate at Station 2 generated by the coordinate transformation matrix *Trt*. *R* is the rotation matrix, *r_11_*~*r_33_* are the rotation parameters, T is the translation matrix, and (*x_0_*, *y_0_*, *z_0_*) are the translation parameters.

**Figure 9:**
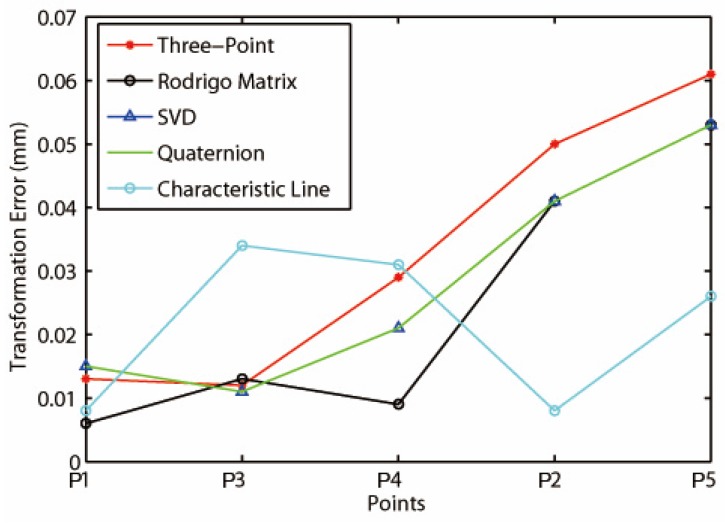
Transformation error compared with different algorithms.

Compared with the actual data measured by the laser tracker in Station 2, the error of the coordinate transformation can be obtained. We also use the root mean square (RMS) of the transformation error of the five public points to describe the transformation accuracy. The experimental results are shown in Table 4 and Figure 9.

The following conclusions can be drawn by the comparison of the results with different algorithms. The accuracy of the Three-Point method is the lowest and its solution depends on the choice of public points. But its algorithm is simple and has the lowest time cost. The Rodrigo Matrix method has the highest accuracy, but the computation of the matrix might be the most complex. It also takes a long time for the calculation. The accuracies of the Singular Value Decomposition method and the Quaternion method are relatively high. The calculation of matrix in the two algorithms are simple and time saving. But they may not be able to work out the rotation matrix when the points are dense. The Characteristic Line method has the same level of accuracy as the Singular Value Decomposition method and the Quaternion method. Its algorithm are simple and stable, and its execution time is short as well. In addition, as Figure 9 shows, it can suppress the large error terms of the other four methods, and does not cumulate errors with the increase of the number of the common points like the other four methods do.

## 5. Conclusions

This paper proposes a simple method of coordinate transformation in a multi-sensor combination measurement system for use in the field of industrial robot calibration. It does not require a large amount of computation or a large field of view and is not affected by the errors of the robot. It operates fast, therefore, it can be integrated into the host computer program. It can be applied in cases where the coordinate systems change often. As verified by experiments, the accuracy of the transformation method is 10^−3^ mm. It reduces the cumulative error of the coordinate transformation in the robot calibration, thereby improving the accuracy of the robot calibration. To evaluate its performance, the accuracy is compared with four common coordinate transformation methods. The experimental results show that the proposed method has the same high accuracy as the Singular Value Decomposition method and the Quaternion method. It can suppress large error terms and does not cumulate errors. Therefore, this method has the advantages of being simple and fast and exhibiting high accuracy and stability. It should have a wide range of application in the field of industrial combination measurement.

## Figures and Tables

**Table 1:** Calculated Results of Coincidence Parameters (Units: mm, °).

Robot to Photogrammetric System						Robot to Laser Tracker					
**a_1_**	b_1_	c_1_	α_1_	β_1_	θ_1_	a_1_	b_1_	c_1_	α_1_	β_1_	θ_1_
−2588.9	81326	−62472	127.53°	1.4461°	256.282°	−3.0135	11.132	−5.8921	117.89°	13.456°	0.007°
**a_2_**	**b_2_**	**c_2_**	**α_2_**	**β_2_**	**θ_2_**	a_2_	**b_2_**	**c_2_**	**α_2_**	**β_2_**	**θ_2_**
−30491	−39586	1397.2	87.979°	37.588°	208.161°	−6.143	0.26899	−5.9267	177.4°	45.997°	0.004°
**a_3_**	**b_3_**	**c_3_**	**α_3_**	**β_3_**	**θ_3_**	a_3_	**b_3_**	**c_3_**	**α_3_**	**β_3_**	**θ_3_**
210.37	−155.16	194.69	38.555°	40.198°	176.953°	200.03	159.99	−200.07	141.35°	37.984°	0.005°

**Table 2:** Coordinates of Point clouds after Transformation (Units: mm).

Robot to Photogrammetric System						Robot to Laser Tracker					
Photogrammetric system			Robot			Laser tracker			Robot		
P_x_	P_y_	P_z_	R_x_	R_y_	R_z_	L_x_	L_y_	L_z_	R_x_	R_y_	R_z_
42.728	138.567	109.566	895	30	875	1048.620	29.944	875.077	895	30	875
108.751	140.724	108.418	961	30	875	1114.679	29.985	874.995	961	30	875
175.846	143.007	107.153	1028	30	875	1181.646	29.955	874.989	1028	30	875
242.882	145.388	105.845	1095	30	875	1248.689	29.935	874.791	1095	30	875
76 points are ignored						76 points are ignored					
Transformation result			error			Transformation result			error		
T_x_	T_y_	T_z_	Δx	Δy	**Δz**	T_x_	**T_y_**	**T_z_**	**Δx**	**Δy**	**Δz**
42.975	138.590	109.751	−0.247	−0.023	−0.185	1048.624	29.967	875.024	−0.004	−0.023	0.053
108.921	140.822	108.277	−0.170	−0.098	0.141	1114.625	29.956	875.012	0.054	0.029	−0.017
175.866	143.088	106.780	−0.020	−0.081	0.373	1181.624	29.944	875.001	0.022	0.011	−0.012
242.812	145.355	105.282	0.070	0.033	0.563	1248.624	29.932	874.890	0.065	0.003	−0.099
76 points are ignored						76 points are ignored					

**Table 3:** The RMS of position error calibrated in the different regions.

Region	O	O_1_	O_2_	O_3_	O_4_	O_5_
Position error/mm	0.200	0.330	0.360	0.271	0.335	0.319

**Table 4:** RMS of transformation error with the different algorithms.

**Points**	**Station 1**			**Station 2**		
	**x/mm**	**y/mm**	**z/mm**	**x/mm**	**y/mm**	**z/mm**
1	3049.626	−188.668	−1403.555	1484.68	1639.268	−1401.164
2	4247.93	991.939	−1401.334	1050.101	3264.365	−1396.089
3	1678.935	1946.842	−1380.022	−1049.19	1502.397	−1379.453
4	3688.375	2777.637	−1403.824	−778.88	3659.965	−1398.95
5	3802.578	1207.190	−1397.241	642.931	2983.472	−1392.788
**Points**	**Three-Point**	**Rodrigo Matrix**	**SVD**	**Quaternion**	**Characteristic Line**	
	**RMS/mm**	**RMS/mm**	**RMS/mm**	**RMS/mm**	**RMS/mm**	
1	0.013	0.006	0.015	0.015	0.008	
2	0.050	0.041	0.041	0.041	0.008	
3	0.012	0.013	0.011	0.011	0.034	
4	0.029	0.009	0.021	0.021	0.031	
5	0.061	0.053	0.053	0.053	0.026	
$\bar{RMS}$	0.033	0.024	0.027	0.028	0.025	
Execution time/s	0.021	0.203	0.031	0.023	0.029	
